# Complicity in Moral Injury: A Study of Moral Phenomena in Healthcare Work Settings

**DOI:** 10.2147/JHL.S568758

**Published:** 2026-04-22

**Authors:** Tracey Rosell

**Affiliations:** 1 Department of Health & Community Sciences, Faculty of Health & Life Sciences, University of Exeter, Exeter, Devon, UK

**Keywords:** command, healthcare, logic of care, moral injury, war-mode leadership

## Abstract

**Introduction:**

Moral injury is characterized by profound, potentially fatal, emotional distress due to perceived violations of moral beliefs. This article addresses calls for improved conceptual clarity about the causes, prevention and treatment of healthcare workers’ moral injury. The dominant focus historically in military and psychological studies was on moral injury arising from isolated events. Recent healthcare organization studies have expanded this focus to consider moral injury as a cumulative phenomenon and to emphasize the need to develop greater understanding of causes, prevention and treatment of healthcare workers’ moral injury. Extending these themes, this article explores, first, if altering a mode of leadership contributes to moral injury occurring, and second, the need to distinguish cumulative moral injury from “lower-consequence” moral phenomena. The question posed is: “what intervention points exist at which leaders could address lower-consequence types of moral phenomena, thereby preventing moral injury occurring?” This involves consideration of a range of phenomenon, including moral traps, demoralization, moral conflicts, moral dilemma, moral stress, moral distress, and moral suffering.

**Methods:**

This qualitative study investigates moral injury and its analysis of interviews focusing on the reported changes in organizational leadership that shaped the experiences of 71 UK National Health Service healthcare workers.

**Results:**

The outcomes of the study contribute to the understanding of moral injury in two main ways. One is by opening the black box on moral phenomena, it became possible to theorize that what some workers report as moral injury was one of several less extreme forms of moral phenomena with less severe consequences. The second contribution reveals why during an atypical crisis the change in the mode of leadership, mandated by a healthcare organizational policy, appeared to be complicit in the reports of moral injury occurring.

**Discussion:**

The discussion concludes by advocating leaders develop a nuanced understanding of the pathways between moral phenomena, and the intervention points at which lower-consequence types of moral phenomena could be addressed, thereby preventing their transition to the more extreme phenomenon, moral injury.

## Introduction

Described as devastating and disorienting, moral injury is the strong emotional response to an act, event, or omission which violates a person’s moral beliefs or expectations.^1^,^2^ It arises when someone experiences a betrayal of what they consider morally right, whether by a figure of legitimate authority or their own (in)action. The emotional, psychological, behavioural, spiritual, and social effects of moral injury are long-lasting, preventing trust and potentially leading to despair, violence, and suicidality.^3^ Traditionally, particularly in military contexts, studies focus on moral injury arising from isolated morally injurious events (MIE), which are events causing moral injury, and potentially morally injurious events (PMIE). They tend to emphasize emotional and psychological causes and impacts. This study differs in two ways in order to contribute to advances in the field of moral injury. First, it considers whether a change in the mode of leadership may constitute an MIE. Second, it addresses developments in recent studies in healthcare organizations that are shifting the perception of moral injury as isolated events. Researchers have indicated moral injury can arise on a cumulative basis and also started to develop greater understanding of the potential methods to address moral injury experienced by healthcare workers.^4^,^5^ In response, this article explores the complex pathways to moral injury. It engages with the need to distinguish moral injury from “lower-consequence” phenomena associated with moral injury.^6^ To pursue this, phenomena considered here include moral traps, demoralization, moral conflicts, moral dilemma, moral stress, moral distress, and moral suffering. Collectively, these phenomena are referred to below as “moral phenomena”. The question posed is: “what intervention points exist at which leaders could address lower-consequence types of moral phenomena, thereby preventing moral injury occurring?”

Since 2020, a significant proportion of UK healthcare staff have reported exposure to morally injurious events.^7–9^ Moral injury, however, is not confined to the UK or the Covid-19 pandemic: reports indicate that healthcare personnel worldwide suffer from moral injury.^10–12^ It also extends beyond healthcare to professions such as veterinary medicine and teaching, as well as various business and non-commercial settings.^13–16^ Since academic studies of moral injury emerged in the 1990s, annual publications increased from about 50 before 2016 to over 300 since 2023. Although U.S. studies dominated early research—comprising 75% of moral injury studies from 1992–2015—this share fell below 55% in the following decade as contributions diversified geographically. While no comprehensive international comparison of moral injury exists, research on moral judgment and harm from unethical behaviour has shown “reasonably robust” findings across different populations and countries.^17^(p82)

Historically, moral injury studies have focused on psychology, the military, and clinical settings, where “clinical” denotes work in patient treatment and care. This article instead examines healthcare workers’ experiences as employees, not as patients, by drawing on interviews from 2020 to 2025 with 71 UK National Health Service (NHS) staff. This study considers whether moral injury results from recurrent experiences rather than a single MIE and proposes that changes in leadership modes can trigger lower-consequence moral phenomena ultimately leading to moral injury. Since 2009 instruments to measure exposure to PMIEs and moral injury have been and continue to be designed,^18^ which will support clinical practice and treatment. However, this study addresses the subject not from a clinical perspective, but from the perspective of a healthcare leader, manager or policymaker seeking greater understanding of issues, and how the organizational policies and their own (in)actions are impactful in colleagues’ experiences of moral phenomena including moral injury.

The context of the original study was the NHS’ response to the Covid-19 pandemic (“the emergency”), involving its fastest-ever national, regional, and local repurposing of standard operating procedures.^19^ During the early stages of the Covid “invisible mugger”,^20^ NHS leaders were advised to communicate frankly, reinforce individual roles, and strengthen social bonds to protect frontline staff’s psychological wellbeing.^21^ However, studies indicated that organizational changes strained working relationships between senior leaders and workers, and without honest, timely communication, contribute to moral injury.^1^

Part of this NHS response was to activate an established command framework (“command”): its implementation on this occasion differed from prior emergencies. Specifically, it was enforced over a prolonged period and incorporated a war-mode of leadership—a system designed to disrupt existing structures to neutralize an “enemy”.^22^ It is proposed that this shift in command operations contributed to moral injury, as the war-mode conflicted with the logic of care that underpins clinical work. Logics are “sensible methods of thinking and making good decisions”.^23^ The logic of care centres on providing medical and social support in ways that help patients navigate illness, considering them as individuals within a social context rather than merely diseased bodies.^24^ In education, the ethics of caring and care ethics highlight moral obligations arising from empathetic engagement.^13^ Though beyond this article’s scope, these concepts are referenced to differentiate the logic of care, which emphasizes the *method* of providing care, as well as relational and ethical aspects of caregiving which are central to ethics of caring and care ethics.^25^,^26^

This article is structured as follows: first, the theoretical foundations of moral injury are presented and its relationship to other moral phenomena. Next, the study’s methodology is described, including data collection and analysis of interviews and observations of 71 clinical and managerial staff. The findings illustrate how moral injury was perceived when the war-mode permeated healthcare operations, sustained by senior leaders even as the emergency subsided. It is revealed how these shifts in command operations altered interactions between senior leaders and workers, contributing to perceived violations of moral expectations. The analysis, informed by empirical data since the onset of the Covid-19 pandemic, advances the study of moral injury and its “darker consequences”.^27^ It is shown that senior leaders’ (in)actions were perceived as lacking a moral basis by frontline workers, particularly when the war-mode of leadership impeded workers’ ability to adhere to their moral logic of care. Additionally, workers felt they had betrayed their *own* moral code by prioritizing the war-mode in their work.

The discussion and conclusion focus on two key themes. First, moral injury can result from systemic moral traps and overstressed systems caused by policies and modes of leadership: that is, moral injury does not necessarily arise from *isolated* MIEs. Moral traps are situations created by systemic, policy and societal arrangements that pressurize workers to act contrary to what they consider is right.^28^ If unresolved, the effects of these can transition into moral injury. Second, distinguishing between different moral phenomena is essential: recognizing the pathways between lower-consequence moral phenomena and moral injury affords the opportunity to mitigate moral traps and overstressed systems. Consequently, there is the possibility of preventing different forms of moral phenomena transitioning to moral injury.

## Moral Injury

The concept of moral injury as psychological harm from violating deeply held moral beliefs dates back to ancient Greek literature and forms the foundation of military studies.^2^,^29^ A potentially morally injurious event (PMIE) may, depending on an individual’s response, become a morally injurious event (MIE) and lead to moral injury. Shay^3^(p182) defines MIEs occurring when there is a betrayal of “what’s right” by a legitimate authority, or oneself, in a high-stakes situation.

Moral injury is a strong emotional response to an act or omission violating a person’s moral beliefs or expectations.^1^ The violation element includes physical harm, such as rape, but also extends to non-physical breaches, including the infringement of rules, codes, or principles (Oxford English Dictionary). This article refers to this non-physical violation when discussing moral injury’s effects, linked to mental health conditions, posttraumatic stress disorder (PTSD), depression, and suicidality.^30^ Documented violations include deliberate negligence, mistreatment, and betrayal, such as failing to support workers,^9^,^31^ policies contributing to unemployment,^32^ and business frameworks for healthcare impeding prioritization of healing over profit.^33^ PTSD is extensively debated in the military and psychological literature. Whilst it may seem the same, it is distinguished from moral injury by clinicians.^30^,^34^,^35^ It is important to acknowledge debates regarding the association between moral injury and PTSD, but these are outside the focus and capacity of this article.

Healthcare studies have identified moral injury as a multidimensional effect, resulting in moral emotions such as anger, guilt, depression, and anxiety.^36^ Emotions are relational, messy and spontaneous, yet can be suppressed, shaped by morals, values, attitudes, and dispositions.^37^,^38^ Strong emotions—both positive and negative—arise from events in work settings and drive action, but negative emotions do not necessarily lead to negative outcomes.^39^ While these studies of emotions, emotional work, and emotional labour inform understanding of strong emotional responses, research on moral injury in non-military settings remains limited.^40^,^41^

Military studies are markedly more extensive, assessing MIEs in combat. They offer some insights for other contexts, linking moral injury to breaches in social moral contracts.^31^ Theorization extends beyond life-threatening events to violations of moral beliefs by leaders or individuals themselves. However, while the military field offers an established understanding of moral injury, its definitions, empirical evidence, and conceptual consistency remain problematic.^42^ People struggle to articulate moral injury with specificity,^43^ and there is a lack of empirical studies on whether organizational changes, including leadership, in non-military settings cause moral injury.

### Moral Phenomena Related to Moral Injury

Thus, despite increased attention to moral injury in organizational contexts, its relationship to related moral phenomena is underexplored. This article explores moral injury in work settings contexts to address these gaps, using Sugrue’s^44^ proposed conceptual model of moral suffering as a start point. This article expands on that model to examine moral injury in the context of work settings, by developing the notion of moral phenomena’s cyclical and reciprocal nature. The term “moral phenomena” encompasses various issues found in work settings, as defined in Table 1. Based on this theoretical perspective, this article considers, first, whether what workers identify as moral injury is actually a different phenomenon, and second, with that knowledge, whether there are opportunities to resolve the phenomena’s effects, before they transition into moral injury.

**Table 1:** Definitions of Moral Phenomena for Work in Organizations

Phenomenon	Author(s)	Definition	Distinguishing Characteristic(s)	Effects/Consequences
Moral traps	Oser^28^	Situations created by systemic, policy and societal arrangements that pressurize workers to act contrary to what they consider is right.	Resources, policies and organizational arrangements	May result in moral dilemmas and conflicts and subsequently moral injury.
				
Demoralization	Clarke & Kissane;^45^ Gabel^46^	Feeling unable to deal appropriately and effectively to stressful experiences. Inability to access moral rewards of work.	Reaction to threat to or loss of personal/professional values related to personal sense of wellbeing.	Feeling impotent, isolated, anxious, depressed, despairing.
				
Moral conflicts	Oser^28^	Tension due to inability to meet three moral claims: justice, care, truthfulness.	Sources may be at structural, institutional/social/individual levels.	Moral stress
				
Moral dilemma	Lissman et al^13^		No clear-cut decision possible	Unresolved moral dilemmas may result in moral stress and consequently moral injury
				
Moral stress	Cribb;^47^ Lissman et al^13^	Foreseeable effect due to overstressed systems, which interferes with workers performing duties according to their preference. Stressors may be during normal, and not necessarily in crisis contexts.	The system creates stressors. Not restricted to patient care. Worker not necessarily powerless to address stressor.	Does not necessarily lead to distress. Manifestation of stress differs from person-to-person. Unresolved moral stress may result in moral distress and moral injury.
				
Moral distress	Jameton^48^ Čartolovni et al^10^	Emotional response to specific event(s) which interferes with workers carrying out duties according to their preference.	Tends to be caused by institutional obstacles, then person’s emotional response to not acting on their initial distress. Sense of powerlessness.	Strong negative feelings eg. anger, shame. Repetitive incidents of moral distress may lead to moral injury.
				
Moral injury	Litz et al^2^ Shay;^3^ Hegarty et al^1^	Response to events, acts or omissions that violates person’s moral beliefs/expectations. Triggered by betrayal of what person considers to be morally right, by an authority figure or their own (in)action.	Strong emotional response to MIE. Creates deep, lasting emotional wound.	Devastating and disorienting effect: can prevent trustfulness, and cause despair, suicidality, and violence.
				
Moral suffering	Sugrue^44^	An integrated model that includes moral distress, demoralization and moral injury.	Model describes circular and reciprocal relationship between immoral actions and immoral contexts.	Individual immoral actions produce immoral contexts and vice-versa.
				

## Materials and Methods

### Research Setting

**Figure 1:**
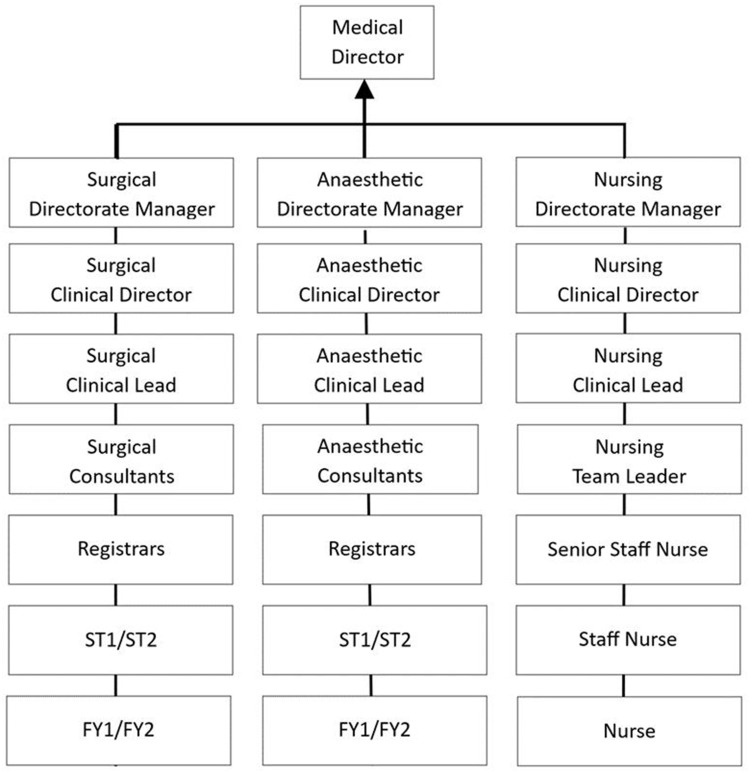
Example of a clinical management chain. Source: author.

The larger project that this article comes from had the overarching aim to explore what changes in leadership members of NHS surgical teams experienced since the 1980s. The unexpected arrival of the Covid-19 pandemic threw certain issues into the spotlight. The first issue was the sudden change in the way a longstanding policy was operationalized. In order to care for patients effectively during disruptive “major incidents”, such as terrorist attacks and winter peaks in demand for hospital stays, the NHS uses an emergency command and control (“command”) framework. Command activities are deployed in accordance with an NHS policy^49^ (“NHS Emergency Framework”). Command’s roots lie in the military where command leadership follows a forked, multi-layered process (Figure 1). Command emerges from a central point from which it is coordinated and may be controlled when required, but principally is decentralized and delegated in a structured way. Nonetheless, it is adaptable, allowing a more informal networked structure to emerge if the situation requires it.^50^

**Figure 2:**
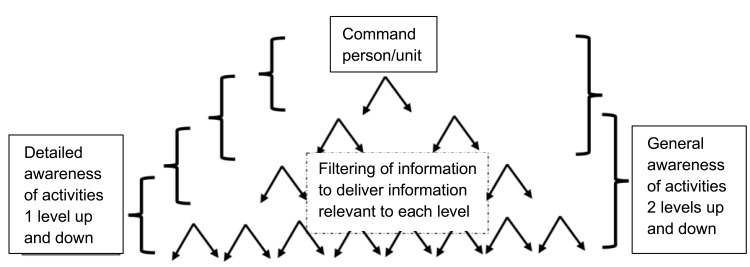
The command multi-layered process. Source: author.

**Note:** arrows denote the filtering down of information to other levels.

This contrasts with how command can be used in the NHS, because the NHS’ linear organizational structure does not naturally support the networked, decentralized operational aims of military command. While it may vary to some extent from one NHS site to another, the clinical leadership and management hierarchy are predominantly linear, rather than forked (Figure 2). The three clinical specialisms, surgical, nursing and anaesthetics, do not have a mutual point in their reporting lines until they reach the Medical Director. Thus, while the military model and the NHS policy model carry the same nomenclature of “command”, they are contrasting models.

### The Experience of Moral Phenomena During the Protracted Exercise of Command

The second issue that came to the fore was moral injury. From an early point in data collection moral injury featured in participants’ contributions, sometimes prominently. They described vividly the events leading up to and the intense experience of moral injury. The researcher was curious about apparent links between the use of command, within an organization whose purpose is to care for people, to produce a strong, negative emotional effect on its workers. Another aspect that was interesting was that this study was based on a long-lasting context underpinning the strong emotional impact on the study’s participants, which contrasted with PMIEs in previous studies, which tended to be sudden, sharp occurrences.^51^,^52^

The crisis caused by the Covid-19 pandemic was “like no other that most healthcare workers have been exposed to during their careers or lifetimes”.^53^(p1) Surveys conducted during the pandemic reported 36% of nurses (increased from 27%) and over 30% of doctors (more than double the previous year) were considering leaving the profession.^54^,^55^ The contributory factors for leaving may be complex and individual, including factors such as political interventions, lack of resources, fear of an unknown disease and the impact on healthcare workers’ family safety. However, evidence indicates the use of command also contributed to individuals’ decision to leave.^56^ Command was retained for substantially longer than normal. It was said to be “the worst thing” for workers ability to perform in a professional manner and for their wellbeing.^56^(p5) Wellbeing is more than the absence of ill-health but may be influenced by physical and mental health. It denotes people feeling their lives are meaningful, satisfying and positive emotionally.^57^

The complexity of the healthcare context led to the choice of a qualitative approach. The study drew on the experiences of changes and emerging dynamics in three NHS hospitals located in urban locations in England and Wales. One belongs to an NHS Trust based in England (“Hospital E”) and the other two NHS Trusts are based in Wales (“Hospital W1” and “Hospital W2”). Hospital E serves approximately one million residents. It is comprised of three hospitals and employs more than 15,000 staff. Hospital W1 employs over 13,000 staff and is comprised of two large district general hospitals, providing acute surgical and medical services and two local general hospitals, supported by several community and mental health hospitals and day care premises. Hospital W2 provides primary community and hospital services, including acute surgical and mental health services. It employs over 15,000 staff at four hospital sites.

The qualitative approach used is underpinned by a social constructionist epistemology. This enables a close engagement with what is studied, using “why” and “how” questions to develop theory. An emergent case study approach was used to allow for flexibility in dealing with unexpected events in extreme contexts^58^ as the pandemic progressed.

### Data Collection and Analysis

Data collection occurred during the Covid-19 pandemic when there was a naturally occurring omnipresent sense of urgency, panic and fear amongst healthcare workers.^59–61^ This was evident from reports in the national press.^62^,^63^ The interview pool comprised seventy-one current and recently retired NHS employees (clinical, and non-clinical in Executive, Management and support roles) and private practice practitioners. Clinical participants were drawn from different professions (Operating Department Practitioners, Nurses, Nurse Practitioners, Surgeons, Anaesthetists), specialties (General surgery, Emergency surgery, Colorectal, Obstetrics and Gynaecology, Vascular, Anaesthetics, Plastic and Orthopaedic). The career stages of participants varied from practitioners who are professionally qualified but are undergoing training in a chosen surgical specialty (“trainees”), to staff who are experienced in their field (2<5, 5<7, 7<10, 10<15, 15 or more years post initial medical qualification). Table 2 provides details of participants featured in this article.

**Table 2:** Key Participants Featuring in This Article

Pseudonym	Role	Specialism	Gender	Experience (Years)	Interviews
Seb	Consultant Surgeon	Colorectal	Male	15+	2
Parv	Manager	N/A	Male	N/A	1
Alec	Consultant Surgeon	Colorectal	Male	15+	1
Stefania	Nurse	Colorectal	Female	15+	1
Anika	Nurse	Colorectal	Female	15+	1
Sunita	Consultant Surgeon	Colorectal	Female	15+	2
Alex	Consultant Anaesthetist	Anaesthetics	Male	15+	4
Mo	Surgical Registrar	Colorectal	Male	10>15	1
Neil	Surgical Registrar	Emergency	Male	7>10	2
Siri	Surgical Registrar	Multi-disciplinary	Female	5>7	1
Therese	Retired nurse - Trainer	Leadership training	Female	>15	1
Daniel	Surgical Registrar	General surgery	Male	10>15	2
Max	Surgical Registrar	Endocrine	Male	10>15	1
Thierry	Operating Department Practitioner	Multi-disciplinary	Male	15+	1
Diana	Nurse	Colorectal	Female	7>10	1
James	Surgical Registrar	General surgery	Male	10>15	1
Gitta	Nurse	General surgery	Female	15+	1
Inge	Nurse	Colorectal	Female	15+	1
Peter	Consultant Surgeon	Hepato-biliary	Male	15+	1
Hugo	Clinical Manager	N/A	Male	N/A	1
Anna	Operating Department Practitioner & Manager	N/A	Female	15+	1
Rohan	Anaesthetic Registrar	Anaesthetics	Male	15+	1
Nick	Manager	N/A	Male	N/A	1

The original research plan was for rich, in-depth case studies at two NHS hospital sites to be conducted using multiple sources of data. However, when the NHS suspended non-Covid-19 research, the research plan was adapted. This included several steps to enhance triangulation and validity of the data in the absence of field observations. These aimed to counter the effect of self-reported activities and perceptions that may have been taken for granted by staff. As video conferencing was rapidly adopted, observations through virtual media became possible. This provided the opportunity for triangulation of interview data. Additionally, wherever possible, the opportunity was taken to ask participants, on an anonymous basis, about issues that other participants had raised, to test the validity of accounts of experiences. Questions were asked in a naïve way, for example, “How did that make you feel?” and “Why do you think this happens?” Using this open question style, the aim was to obtain genuinely corroborative, or contradictory, perspectives that were not influenced by the researcher or earlier data. If they said they had corroborative experiences, participants were asked to give concrete examples of what they witnessed. This enabled theoretical and practical insights, despite a lack of observations in the field.^64–66^

Eighty-two semi-structured interviews were conducted, a method suitable for exploring relational experiences and “real-world impact” in healthcare.^67–69^ The interviews were carried out over four-and-a-half years, usually lasting 50–75 minutes. Field notes from virtual observations of training and meetings recorded additional insights into features of work and social interactions.

**Figure 3:**
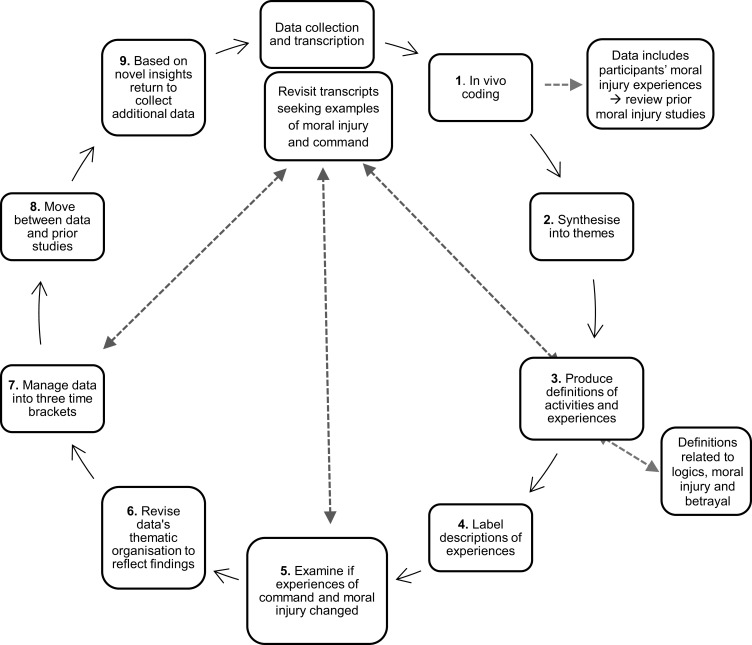
Iterative methodological approach. Source: author.

The analysis conducted comprised of nine stages, adopting a “methodological bricolage” to address the research objectives and facilitate trustworthiness^70^(p211) (Figure 3). The interviews often produced a broad account of experiences, resulting in 52 themes identifiable during the first stage of analysis. Work was carried out to identify overlaps, eliminate ambiguously defined themes, and to identify salient, interesting and/or paradoxical themes that warranted further thought and investigation. The data analysis was iterative, using a cyclical progressive approach rather than a linear fixed sequence.^71^

*Stage 1* of the process was “in vivo” coding of data line-by-line. This comprises creating codes directly from the data, to keep concepts as near as possible to participants’ terminology.^72^,^73^ The data was synthesized into themes in *Stage 2*, based on existing studies. This produced “patterns of shared meaning underpinned or united by a core concept”.^74^(p593) The individual items were mapped onto main themes and then organized into sub-codes to reflect the full variety and meaning of participants’ experiences.^75^,^76^ The transcripts were revisited to look for examples of (1) different types of moral injury; (2) experiences of command; and (3) the intersection of command and moral injury. For *stage 3* of the analysis, working definitions were produced of activities and experiences that participants described. In *stage 4*, based on the data, if possible, participants’ descriptions of experiences were labelled as logic of care, war-mode of leadership, betrayal by person-in-authority, and betrayal-by-oneself. *Stage 5* was an examination of whether people’s experience of moral injury had changed. In *stage 6*, the organization of data from themes was revised to reflect the findings. For *stage 7* data was managed relevant to the time brackets. Experiences were across three successive periods before the pandemic, command activation, and after the first peak of Covid-19 abated.

*Stage 8* involved moving between the data and previous studies concerning moral injury, specifically previous theory concerning cumulative moral injury.^1^,^5^,^77^ It became apparent that there was a weak theoretical understanding of the interplay of command in healthcare organizations and moral injury, particularly regarding cumulative experiences. For *stage 9*, the interview protocol was enhanced to collect additional data to build on initial novel insights and the connection between command and moral injury. Stages 1 to 8 were repeated with new data as additional participants came forward.

## Results

### The Conflict Between the War-Mode and the Logic of Care

The reported widespread panic and fear due to the Covid-19 pandemic was reflected in participants’ accounts of how they felt at the outset of the pandemic.

At the beginning of the pandemic everyone was really scared and understandably so. [Siri: surgical Registrar]

Senior leaders and managers contributed to the formation of a warzone atmosphere. This was through their adoption of terms such as “on the frontline” and a “War Room” for meetings; also, actions such as NHS England advertising that it was “rallying the troops” for the war on coronavirus” as it sought volunteers.^78^ This initial atmospheric work was reinforced by bringing the military into hospitals, which generated an apocalyptic, warzone atmosphere.

I went in for some training in the hospital, that had all kind of closed down. And the army were in there trying to set up there. And I think it was quite apocalyptic feel. It was a palpable, apocalyptic feeling. [Stefania: Nurse]

The organizational context was characterized as a warzone. This was reflected in how people expressed their experiences during the first Covid-19 wave, being “like fighting a losing battle” [Anika: Nurse]. The atmosphere was one of “asymmetric warfare” [Mo: surgical Registrar]. The continuing use of military terms by senior leaders built on and developed this atmosphere of fear in the early stages of the pandemic.

In the eyes of participants, there was a contradiction in terms of the physical and emotional aspects of the war-mode engaged by senior leaders. On the one hand, in terms of physical resources, including equipment and the redeployment of personnel, there was a “knee-jerk reaction from a lot of the health boards…of “Crikey, we must throw all of our resource behind this.” [Mo: Surgical Registrar]. While there was a drive for the provision of PPE, highlighted in the press, less publicized omissions were happening:

Management knew that my working area was not suitable within the Covid period. We, actually in the office that we were in, we had stool and urine leaking through the roof, the ceiling from Intensive Care [where patients with Covid were cared for] into our office. I wasn’t looked after, and neither were my colleagues. [Inge: Nurse]

Such leadership was identified as “not having that basis or foundations of, you know, morality” [Neil: Surgical Registrar]. There was a deficit of emotional resources: senior managers stopped providing support, by failing to empathize with the mental health and wellbeing issues experienced by surgical team-members. This was accentuated by the absence of senior managers at the frontline. They were not there in person to ask, “How are you?” Surgical team-members wanted leaders to,

be seen, be doing, and in those moments when you’re role-modelling, that’s your time to build relationships with those staff. I think the senior staff’s absence was very negative in terms of their relationships with their colleagues. [Gitta: Nurse]

The cumulative emotional effect on participants was stark when participants had the opportunity to collectively present their belief in the logic of care: clinicians described the use of their own values as a basis for this logic which underpinned their work before and during the emergency: “in terms of ethical and moral concepts moving forward, we’re trying to do the most we can for the most patients” [Alex: Consultant anaesthetist]. Working in the surgical environment individuals “acknowledge and act according to their own moral core values. Morally I do work to what I believe is right” [Thierry: ODP]. They believed their own moral standards set, “the bar about, what good and bad behavior or better and worse behavior and decisions are” [Parv: Senior Leader].

The logic of care incorporated the care of workers’ wellbeing too because, particularly in surgical teams, senior personnel thought of colleagues as

not my staff. They’re people I work with, my colleagues, as well. But you think of them like your family. And that’s the difference between, I suppose, the Theatres and the ICU teams is we tend to think ourselves, of ourselves as a family. [Alec: Consultant anaesthetist]

During the emergency, the logic of care informed the Consultants’ belief that the Theatres could not continue to be run safely. Initially, their combined voice was ignored by senior leaders. Eventually, with middle-level manager intervention, they managed to give their clinical opinion: the manager’s intervention was crucial to gain access to senior leaders exercising command. Senior consultants were transformed from their usual pre-pandemic habit of “sit[ting] in the background chuntering” during meetings. Instead, together they gave such a display of raw emotion about the clinical need to close Theatres that “there wasn’t a dry eye in the house” [Anna: Clinical Manager].

As the pandemic progressed, a clinical view developed that while parts of the hospital had to be dedicated to the treatment of Covid, the leaders should have dedicated part to the treatment of other medical conditions. Failure to do so meant that operating stopped completely and the consequent fatal impact on patients resulted in a strong emotional response among clinical staff:

We were seeing a lot of cancer patients who, those who had had to delay their treatment, never getting that treatment OK, or it was too late, and they died. And it was. And sometimes this hit us emotionally more than the Covid itself. Knowing that a person was able to, could have had the treatment at the right time. [Rohan: Anaesthetist]

What staff were told to do in their opinion conflicted with ethical and moral values they held for patients’ care, exemplified by Diana et al objecting to senior leaders’ decisions, saying, “This is unsafe, we can’t do this!” [Diana: Nurse]. The ongoing use of command was perceived to have “wrecked” the relationship between those in command or senior leadership roles and people working in and with surgical teams. The effects of violations of those moral values during the pandemic were acknowledged and took many forms.

I do think that’s the thing that Covid has thrown up, is how do we address the moral injury. And you know, all these things that have left health professionals in not a good place. [Therese: retired Nurse]

In hearing the accounts of other strong emotional responses to what happened, “not in a good place” understated the majority of emotional responses to the events, acts or omissions that violated moral beliefs or expectation. Morale was “decimated” and “it made us all feel rubbish. No one felt cared about” [Daniel: Surgical Registrar]. Some thought command was like a “dictatorship”: Consultants were used to being given directions but then having autonomy to communicate and arrange what needed to be done in their own way. This autonomy was removed and left people feeling powerless.

It’s a bit like a Rubik’s Cube, isn’t it? You’re given the puzzle, there are several different ways of solving it. The problem with the command-and-control bit is that you were also given the way to solve it as well. I think that made people, at all levels, feel quite powerless. But you weren’t in control. At a time when you were already very much feeling like you weren’t in control. [Peter: Consultant surgeon]

The detrimental effect of stripping away their professional influence and silencing them by making it impossible to speak up about issues they were concerned about, swelled as the pandemic progressed:

there’s anxiety around loss of control…I’ve heard of people losing autonomy…I didn’t hear anybody that was complaining about not being not being consulted, the first wave. I think those voices got louder in the second wave and now third wave. [Hugo: clinical Manager]

Workers witnessed what they identified as colleagues’ moral injuries, perceiving them as being treated “like pawns in a game” instead of being “more valued as actual human beings” [Diana: Nurse]. People were drafted into areas that they were inexperienced in, often leaving clinicians feeling it resulted in some unexperienced staff being broken, as well as the provision of “sub-optimal care” to patients:

it’s like putting a toddler in the middle of a park and then walking off and leaving them. They just don’t know what to do…. If they’re old enough, they know that you’re going to come back, and they’ll help them, and they can rationalize it. But there’s a group in the middle who haven’t got that knowledge and experience yet and would just completely melt and be broken at the end of it. [Alex: Consultant anaesthetist]

This contributed to morale declining further, “panic responses in hospitals” [Observation note:01] and people feeling “scared”, in turn leading to staff absence, “a massive deficit” through sick leave, resignations and early retirement. Team leaders were “dealing with people having breakdowns, people having panic attacks” day-to-day [Nick: Manager]. It was experienced as “not one sudden moral injury that utterly ruins people. It’s subtle, gentle, constant 400 times a day little moral injuries” [Daniel: Surgical Registrar]. This was seen to endure along the road to Covid recovery, as people adjusted to the “new normal”. Participants considered the experience of seeing “horrific outcomes” for patients affected workers’ view on life, so it was wrong to expect people to switch back into that “normal” without help. Leaders needed to have

an appreciation of the fact that the effects of something like a pandemic can vary hugely depending on what your role is in the team…they were redeployed to intensive care, they’ve gone from watching a lot of people have quite horrific outcomes in the intensive care unit. And then the next day, they’re back in Theatres expected to be, just carry on as normal…I think that we’re looking at people who are damaged. Who were just being shot back into a scenario. Because they’ve been familiar with it previously, they’re expected to carry on, you know, and I don’t think that’s right. [Max: Surgical Registrar]

### Becoming Complicit Oneself: Privileging the War-Mode

People found that their own empathetic values diminished, in their reactions to colleagues’ absences from work. When their critical perspective of colleagues’ absence was challenged, this too felt like self-betrayal of their own collegiality.

What I thought was shirking responsibility and not wanting to come to work when actually a lot of people probably were just shielding. A lot of people were very sick. And a lot of people died. So that was quite inconsiderate of me. Then I had a discussion with one of the SHOs. I think I said something flippant, like, ‘*I can’t believe so many scrub staff have taken off, taken on the sick. How many of them are actually unwell*?” And she was like, “Well, you know, a lot of them are actually probably unwell, James, and a lot of them probably need shield as well because, you know, health issues or whatever, so’. Yeah. I just felt quite bad about myself. I was just like, ‘Oh, yeah, yeah. Terrible’. [James: Surgical Registrar]

The privileging of the war-mode at this time, over the logic of care was not solely the action of senior leaders. In response to war-mode of leadership clinicians changed their habitual way of working with the logic of care. This was associated with people seeing colleagues suffering prolonged stress.

We had two patients that have come to harm, because they have seen, I think it was documented, one person had seen 20 Consultants during their stay…continuity of care means that you see a patient and you can figure out when they’re better or worse, because you saw them the day before. So, we have now gone to, we then went to a system where this was, we felt dangerous…before Covid they were the one banging on about continuity of care. It’s really strange that sort of switch…But it doesn’t fit with the chaotic nature of what’s going on at the moment. And it’s really causing them stress, massive stress and their stress is translating into how they’re behaving with colleagues, how they’re behaving with staff. [Sunita: Consultant surgeon]

After the first peak of the emergency abated, surgical teams engaged in the war-mode of operations too, violating their own professional rubrics. Teams were vying against other teams to compete for resources, such that patients’ treatment by one specialty was reduced due to the actions of another specialist department. Part of the war-mode of leadership was to ban elective surgery (non-emergency, planned operations), to which clinicians found a work-around:

They reprioritized all the patients that were routine and made them urgent, on the basis that they had a cancer. Prostate cancer is a benign cancer, so basically elevated the urgency. So, they were then allowed to continue because their cases are short volume, they did a lot of them. But the problem with that was that it was actually to the detriment of other departments who then couldn’t get priority. [Sunita: Consultant surgeon]

Consultant surgeons began to triage patients, using a selection process that differed from the norm, taking into account the restricted hospital resources available. As would happen on a battlefield, they were “culling” people, deciding who would live and who would die. Where the ban on elective surgery had not yet come into force, clinicians worked to do as many operations as possible before the ban started.

We had 12 cancer patients that needed doing. And we knew this wave was coming of Covid, and it was mid-March. And I don’t know if, you know, we got it really bad at hospital A…We got it bad… So, but we saw them all in clinic, in one clinic. And I, I organized anaesthetists, all of the surgeons. So, we had a big clinic. We culled four or five elderly patients that we would have normally operated on, because we know Covid is coming, we got rid of four or five patients, said, ‘You’*re not having surgery*’. We just did the other eight or nine. [Seb: Consultant surgeon]

## Discussion

Recently organization studies presented moral injury in an alternative light to the historic view of it being caused by an isolated event: evidence was presented of it as a cumulative phenomenon amongst healthcare workers. This coincides with calls, including in this journal, to better understand causes, prevention and treatment of healthcare workers’ moral injury.^4^,^5^ The experiences of the participants in this current leadership study presented the opportunity to extend understanding around these themes. This article arises out of a larger project, additional details of which are provided in Rosell’s^79^ article regarding the liminal duality of expert authority in UK surgical teams.

The findings from this part of that larger project exposed that not all self-reported experiences of moral injury were necessarily that particular phenomenon, and an examination of lower-consequence moral phenomena was needed. The context of the study afforded an insight into how a change in a mode of leadership was complicit in the manifestation of moral phenomena. This was because the effects of the altered leadership conflicted with workers’ embedded moral values. The final contribution of this article is a framework which extends previous study of a small number of moral phenomena grouped together—for example, see VanderWeele et al’s^18^ consideration of moral injury and moral distress. The framework delineates (a) more extensively the continuum that a more comprehensive assemblage of moral phenomena extends along, and (b) the pathways between them that can lead to moral injury. This assists healthcare leaders, managers and policymakers expand their knowledge about moral phenomena, their understanding of pathways to moral injury, and intervention points at which lower-consequence types of moral phenomena could be addressed. Such interventions serve to disrupt the feedback loop Sugrue^44^ proposes between an immoral context and collective individual immoral actions. This results in the prospect of preventing a transition from lower-consequence phenomena to the more extreme phenomenon, moral injury.

### Self-Reported Moral Injury

Based on the findings it is suggested that the (self-)reporting of moral injury and a lack of conceptual clarity from studies to date may be providing inaccurate indications of the moral phenomena experienced in healthcare settings. Moral injury studies have primarily focused on the clinical treatment of military personnel, both serving and veteran, and research has remained siloed within psychology, military, and clinical fields, even though harm can occur in any social context.^80^ In contrast, this study analyzes how workers experienced moral phenomena during a prolonged and atypical emergency, not as clinical patients but from their perspective as workers. It shows that senior leaders’ adoption of a war-mode of leadership conflicted with workers’ embedded logic of care. This led some to perceive the leaders’ and their own (in)actions resulted in moral injury, distinguishable from other phenomena by its resultant deep, lasting emotional wound, leading to loss of hope, trust, and integrity.^2^,^10^ Some workers reported positive experiences during the pandemic of command, particularly where they were given increased autonomy.^81^ However, given other international reports of moral injury among healthcare workers during the pandemic, the types of negative experiences described by the participants in this study were fairly common ones. These experiences included negative reactions to the use of command, including a warzone mode of leadership in a civilian setting. The widespread moral beliefs across the workforce, which underpin patient care, may explain why substantial numbers of healthcare staff report moral injury.^8^,^9^

### Identifying Moral Phenomena

**Figure 4:**
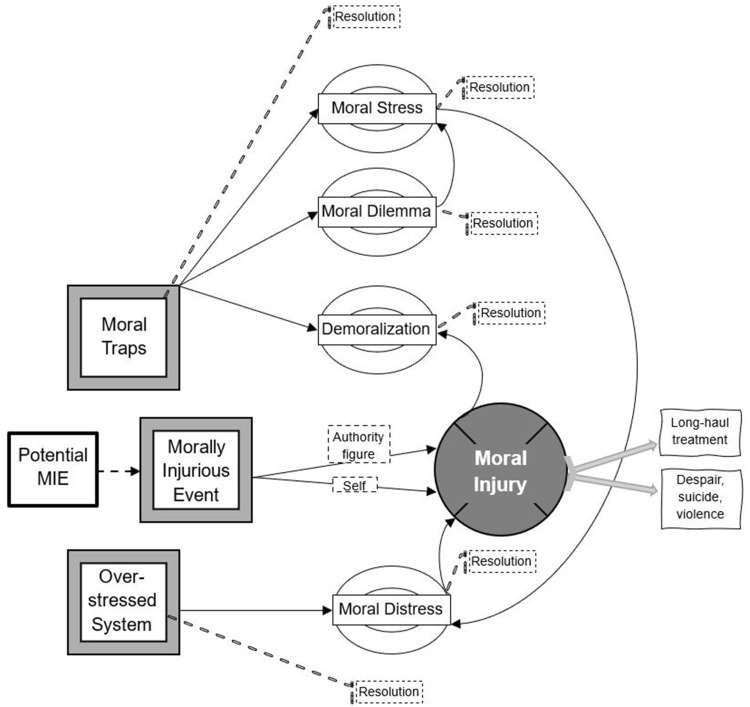
A framework of the moral phenomena continuum and pathways to moral injury. Source: author.

Whilst not suggesting deliberate misreporting of moral injurious experiences, it is proposed here that not all the incidents characterized by the participants as moral injury were correctly identified. Moral injury studies traditionally, particularly in military contexts, focus on isolated MIEs, such as witnessing death, rape, or child soldiers in combat.^82^,^83^ However, recent organizational studies in nursing suggest moral injury can be cumulative.^5^ Reflecting on this and the participants’ accounts of “death by a thousand cuts”, this article explores whether what was experienced in this healthcare work setting was a phenomenon of lower consequence, rather than moral injury, and whether workers’ perceptions align with a cumulative process producing a MIE, or a succession of moral injuries occurring (that are caused by many *isolated* MIEs), or something else. This study opens the black box of moral phenomena in healthcare settings to develop a framework that reflects these possibilities (Figure 4).

Whilst each of those “thousand cuts” may be distressing, they do not necessarily individually or cumulatively amount to the most extreme of the moral phenomena considered here. To identify where the phenomenon experienced sits on the continuum of moral phenomena, needs consideration of two aspects. One aspect, in the absence of a clinical assessment, is to consider whether an individual, organizational, and/or clinical, intervention resolves a moral phenomenon. Successful resolution by amending institutional policies or removing obstacles in work processes which are causing an emotional response could indicate a lower consequence moral phenomenon is in play.^1^,^10^ However, if the only effective resolution transpires to be clinical intervention, this would indicate moral injury is more likely to be the phenomenon experienced.

A proactive assessment of the effects of phenomena may avoid progression to the devastating effects of moral injury and long-term clinical treatment needs.^2^ Nielsen et al^16^ identified the importance of understanding different types of MIEs but it cannot be assumed that the cause or experience resulting in moral injury can be clearly identified.^6^ The assessment of which phenomenon individuals are suffering needs to be undertaken cautiously: in the absence of a clinical diagnosis, it is insufficient for leaders and managers to examine only actions, inaction, values, and policy factors when trying to gauge what phenomenon/workers are experiencing. As Griffin et al^5^ highlight, distinguishing moral injury from other moral phenomena requires examining the feelings the phenomenon creates. Thus, intrinsic in identifying moral phenomena is considering how any of the above four factors, or a combination of them, make people *feel*: for example, the characteristics of effects of moral distress are strong negative feelings, such as anger or shame, whereas the effects of moral injury are experienced as devastating and disorienting, and can lead to despair, suicidality, and violence.

### Intervention Along Pathways to Moral Injury

This contention that leaders need to have a better understanding of the effects of phenomena is addressed by the current study’s contribution to moral injury theory in two ways. First, by offering a nuanced understanding of moral injury and other moral phenomena in a healthcare work context—with moral injury lying at the most extreme end of a continuum of moral phenomena—at a time when moral injury is increasingly relevant to broader society beyond the military and clinical studies. Second, based on this more nuanced understanding, the framework presented is intended to support leadership and policy decision-making, particularly during consideration of enabling recovery following experiences of moral injury and other moral phenomena. While some participants attributed moral injury solely to senior leaders’ (in)actions, their interpretation oversimplifies the issue. The analysis of this study’s data, and the literature on which this framework is based, accounts for moral injury resulting not only from an isolated MIE due to an authority figure’s (in)action, but additionally from policies and processes that create moral traps and overstressed systems. These additional issues can trigger moral phenomena—cumulatively in some cases—which if unresolved may lead to moral injury. The findings demonstrate the pathways to moral injury: an example found in this empirical study is the perceived sudden shutdown of relational, emotional interactions between leaders and frontline workers by virtue of a change in the usual mode of leadership. The NHS Emergency Framework’s policymakers, albeit unintentionally, were complicit in moral injury occurring. This was due to drafting a policy that did not require clinicians’ input during clinical emergencies: the policy does not require senior leaders to consult medical professionals beyond ambulance tactical advisors, omitting terms like medical, clinician, surgical, anaesthetist, or nurse. This lack of consultation drove people’s perceptions of the infliction of moral injury.

Recovery becomes possible only when evidence-based organizational strategies and processes are created which serve to address the source or cause of (potential) moral injury and that will invest in healing the workforce.^33^,^84^ The framework presented here builds on the premise of developing leaders’, managers’ and policymakers’ knowledge of distinguishing characteristics of moral phenomena, their understanding of the pathways to moral injury, plus the potential intervention points.

One illustration of an intervention, potentially an applicable resolution to the warzone scenario in this study, is to use ethical values to explicitly guide future organizational decision-making regarding resource priorities.^85^ Further consideration would need to be given to how values and resource priorities are balanced in atypical situations beyond “normal” crises, and that moral injury is inherently individual.^1^,^3^ As an example, consider Seb and his team’s use of military triage which diverged from care ethics before the pandemic. As Islam^86^(p2) portrays, their actions appeared to be an “attempt to construct new spaces of action, balancing competing motivations to live within imperfect worlds”. In “normal” times, care to ensure individual survival is paramount, whereas in a war-mode, the broader mission and conservation of resources is paramount and dictates triage priorities.^87^ It is theorized here that for some people, this shift in the mode of leadership constitutes an MIE resulting from their own decisions and (in)actions: by complying with the war-mode, some frontline workers experienced a violation of their own moral values. The betrayer of moral values is not only an authority figure but also the individuals themselves. For others, the shift could create a moral trap, which results in negative effects—but ones that could be resolved. Resolutions include alleviating moral distress by adjusting processes, thereby addressing the danger of increased turnover,^88^ addressing systemic causes of moral traps, and improving leadership to counter demoralization.^13^,^46^,^89–91^

Nonetheless, interventions may not always enable recovery, to avoid increased turnover and other effects detrimental to the workforce. As Reynolds et al^92^ observe, “prolonged or poignant levels” of moral phenomena may result in employees giving up on their moral reasoning. This suggests a valve to release the moral (di)stress, as the person no longer finds themselves under pressure to carry out moral reasoning. Alternatively, Reynolds et al propose that there is a counter-intuitive effect: motivation produced by the desire to act morally then contributes to the ongoing moral stress. These two possibilities, labelled for the purposes of this discussion as the “moral release-valve” and “moral-motivation stress”, may explain why some workers remain on pathways to moral injury, due to moral-motivation stress, whereas the moral-release valve operates for others, so the workers feel able to leave behind the logic of care and to exit their job via resignation, sick leave and/or early retirement.

## Conclusion

Despite the potential for harm in any social context, moral injury research remains underdeveloped in non-military work contexts including healthcare. Based on this study’s illustration of how the conflict between modes of leadership and professional values are implicated in moral injury, both directly and indirectly, this article expands on moral injury research. It goes beyond its military and clinical origins to theorize how moral injury unfolds in healthcare work settings, and advancing theory in the field by analyzing moral injury events at two levels: individual, reflecting moral phenomena’s discrete effects, and organizational, highlighting drivers of moral injury—with the aim of identifying points of intervention, to bring about change to an injurious process or (in)actions, thereby preventing or resolving morally injurious pathways and/or events.

With moral injury research expanding beyond psychological, clinical, and military domains, the “invisible wound” of moral injury is increasingly relevant to broader society.^93^ A nuanced understanding is essential as moral injury’s importance in healthcare organizational studies is addressed. While prior research established MIEs can cause sudden moral injury, the framework presented above identifies the slow-burn version, showing how other unresolved moral phenomena can transition into moral injury. As moral injury can arise rapidly or gradually, organizations, leaders and policymakers need to grasp the complexity of moral phenomena if they are to intervene effectively. Addressing policy, systemic, or societal factors contributing to various moral phenomena could enable healthcare leadership to diminish or prevent the incidence of the long-term, potentially fatal harm that moral injury inflicts.

### Further Research

A review of the NHS Emergency Framework is recommended. Given evidence that the current command policy may be implicated in the future moral injury, research could investigate whether an alternative framework—possibly a systems approach combined with ethical leadership—would help prevent or mitigate moral injury. Additionally, knowledge of resolutions for demoralization and moral distress in healthcare and other work contexts is underexplored: future studies could provide organizations with a broader array of resolution options.

Although the definition of moral injury omits the term “ethical”, Griffin et al^5^ describe it as a “betrayal of what is *ethically* right by those in positions of power” (this author’s emphasis). The terms moral and ethical are often used interchangeably. While Shay’s^3^ definition to advance theory at this stage is used in this article, future scholars may explore whether distinguishing these terms offers a more nuanced understanding of moral injury.
